# What is the best prophylaxis against venous thromboembolism in Asians following total knee arthroplasty? A systematic review and network meta-analysis

**DOI:** 10.1186/s43019-022-00166-y

**Published:** 2022-08-13

**Authors:** Soon Yaw Walter Wong, Fen Li Stephanie Ler, Rehena Sultana, Hamid Rahmatullah Bin Abd Razak

**Affiliations:** 1 Department of General Medicine, Ng Teng Fong General Hospital, 1 Jurong East Street 21, Singapore, 609606 Singapore; 2 National University of Ireland, University Road, Galway, H91 TK33 Ireland; 3 Duke-NUS Medical School, 8 College Road, Singapore, 169857 Singapore; 4 Department of Orthopaedic Surgery, Sengkang General Hospital, 110 Sengkang East Way, Singapore, 544886 Singapore; 5 SingHealth Duke-NUS Musculoskeletal Sciences Academic Clinical Programme, Academia Level 4, 20 College Road, Singapore, 169865 Singapore

**Keywords:** Thrombosis, Pulmonary embolism, Haemorrhage, Arthroplasty, Replacement, Knee, Asians, Prophylaxis

## Abstract

**Purpose:**

Asians have a low venous thromboembolism (VTE) incidence following total knee arthroplasty (TKA). This systematic review and network meta-analysis was conducted to evaluate the best prophylaxis against VTE in Asians following total knee arthroplasty in current literature.

**Materials and Methods:**

A systematic search of PubMed, Embase and CINAHL was conducted in adherence with the Preferred Reporting Items for Systematic Reviews and Meta-Analyses (PRISMA). Prophylaxis types were separated into low-molecular-weight heparin (LMWH), novel oral anti-coagulants (NOAC), mechanical-only prophylaxis (MOP) and no prophylaxis (NP). The primary outcome was VTE incidence, grouped according to diagnosis modality (ultrasound, venography, clinical). The secondary outcome was bleeding incidence, grouped into minor and major bleeding.

**Results:**

Fourteen eligible articles, totalling 4259 patients, were pooled with the following significant results: NOACs had lower venography-diagnosed VTE incidence than LMWH (12.77%, *p* = 0.02) and NP (20.64, *p* < 0.001). MOP had lower venography-diagnosed VTE incidence than LMWH (23.72%, *p* < 0.001), NOACs (10.95%, *p* < 0.001) and NP (31.59%, *p* < 0.001) but, interestingly, a statistically higher ultrasound-diagnosed VTE incidence than LMWH (6.56%, *p* = 0.024) and NP (4.88%, *p* = 0.026). No significant differences were observed between prophylaxis types for symptomatic VTE, pulmonary embolism (PE) or death. LMWH and NOACs had a higher minor bleeding incidence than NP (11.71%, *p* < 0.001 and 6.33%, *p* < 0.02, respectively). No significant differences were observed between prophylaxis types for major bleeding incidence.

**Conclusion:**

NOACs are a superior form of chemoprophylaxis, compared with LMWH, in reducing venography-diagnosed VTE incidence with no added bleeding incidence. However, routine chemoprophylaxis may not be required as LMWH and NOACs do not appear to reduce symptomatic VTE incidence compared with MOP and NP with an increased minor bleeding incidence. Mechanical prophylaxis in the form of graduated compression stockings or intermittent pneumatic compression should be routinely considered with significantly lower rates of venography-diagnosed VTE compared with NP. On the basis of current evidence, we recommend an individualised approach to select the most appropriate prophylaxis type.

## Introduction

Venous thromboembolism (VTE), which includes pulmonary embolism (PE) and deep vein thrombosis (DVT), is a significant and well-recognised complication following total knee arthroplasty (TKA). VTE presents a significant public health problem and is associated with the second-highest mortality following total joint replacement [1, 2].

Evidence has shown that VTE rates following surgical procedures including total knee arthroplasty are lower in Asians than in Western populations [3–6]. Some data suggest that genetic polymorphisms are responsible for the lower risk of VTE in Asians [7, 8] With the established low-risk demographic found in Asian countries, many institutions have adopted reduced VTE prophylaxis measures, whereas Western countries largely use routine chemoprophylaxis commonly in the form of low-molecular-weight heparin(LMWH), NOACs or anti-platelets in conjunction with mechanical prophylaxis [2, 9].

Broadly speaking, VTE prophylaxis falls into two categories: mechanical prophylaxis and chemoprophylaxis. Mechanical prophylaxis regimes include early mobilisation, elastic (graduated) compression stockings (GCS) and intermittent pneumatic compression (IPC). Common chemoprophylaxis types include non-steroidal anti-inflammatory drugs (NSAIDs; e.g. Aspirin), novel oral anti-coagulants (e.g. rivaroxaban, dabigatran), heparins (e.g. low-molecular-weight heparin, unfractionated heparin) and warfarin. Both mechanical and chemoprophylaxis have shown effectiveness in reducing VTE rates [10, 11]. However, some reports show that the morbidity and mortality of the haemorrhagic side-effect profile of anti-coagulants may outweigh the benefit of reducing VTE rates [11, 12]. Consequently, when dealing with an Asian population with a lower risk of VTE, further investigation into the risks and benefits of routine VTE prophylaxis is warranted.

Despite the established understanding that Asians have a lower risk for VTE, there is a hiatus in published literature comparing prophylaxis types and a lack of a consensus on the optimal VTE-prophylaxis regime for managing patients after TKA. This systemic review and network meta-analysis aims to evaluate the current literature for the best prophylaxis against venous thromboembolism in terms of efficacy and safety in Asians following total knee arthroplasty.

## Hypothesis

Prior to the initiation of this project, we hypothesised that the rates of VTE would be lowest in chemoprophylaxis, followed by mechanical-only prophylaxis and no prophylaxis. With established lower incidence of VTE in Asian compared with Western populations, we expected a significantly reduced benefit to using chemoprophylaxis over mechanical-only prophylaxis in view of its associated increased bleeding incidence.

## Materials and methods

### Information sources

**Fig. 1:**
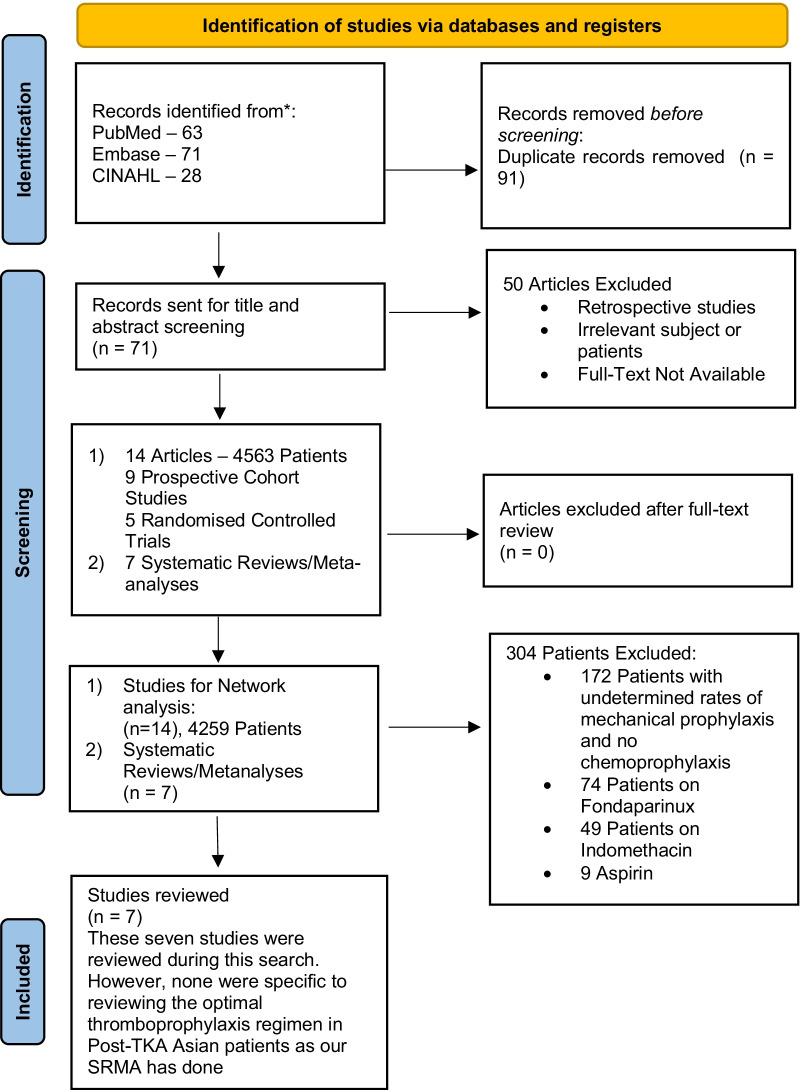
Study flow in accordance with PRISMA 2020

An electronic systematic search was performed by two independent authors (W.W.S.Y. and S.L.F.L) in the PubMed, Embase and CINAHL databases to identify all relevant studies, with the most recent search conducted on 20 June 2022. The following search strategy was used as presented in Table 1: “(Asian) OR (Asians) AND (Total Knee Arthroplasty OR TKA Or Total Knee Replacement Or TKR) AND (Venous Thromboembolism OR VTE OR Deep Vein Thrombosis OR DVT OR Thrombosis OR Pulmonary Embolism OR Anticoagulation)”, for which no date limits were used. Reference lists of relevant systematic reviews were manually searched. A total of 162 articles were identified: 63 in PubMed, 71 in Embase and 28 in CINAHL, of which 71 articles were screened after duplicates were removed. This review was not registered on the PROSPERO database. The search workflow adhered to the Preferred Reporting Items for Systematic Reviews and Meta-Analyses (PRISMA) guidelines [13] and is illustrated in Fig. 1. To identify studies to be included in the final review, the articles were independently assessed by two authors (W.W.S.Y. and S.L.F.L.), to determine eligibility for inclusion in the analysis. Any disagreements were resolved by consensus discussion among the authors. A total of 14 studies were included in the final review. Our search identified no identical systematic reviews or network meta-analyses, as shown in Fig. 1 [13].

**Table 1:** Search strategy

Database	Type of search	Search terms
PubMed database (https://pubmed.ncbi.nlm.nih.gov/) 63 records	Medical subject headings (MeSH) words and general terms	“Asians” [MeSH] OR “Asian” AND “Total Knee Replacement” [MeSH] OR “Total Knee Arthroplasty” OR “TKA” OR “TKR” AND “Venous Thromboembolism” [MeSH] OR “VTE” OR “Deep Vein Thrombosis” OR “DVT” OR “Thrombosis” OR “Pulmonary Embolism”[MeSH] OR “Anticoagulation”
Embase database (https://www.elsevier.com/solutions/embase-biomedical-research) 71 records	Emtree (Elsevier Life Science Thesaurus) and general terms	“Asians” OR “Asian” AND “Total Knee Arthroplasty” OR “TKA” Or “Total Knee Replacement” Or “TKR” AND “Venous Thromboembolism” OR “VTE” OR “Deep Vein Thrombosis” OR “DVT” OR “Thrombosis” OR “Pulmonary Embolism” OR “Anticoagulation”
CINAHL database (https://www.ebsco.com/academic-libraries) 28 records	General terms	“Asians” OR “Asian” AND “Total Knee Arthroplasty” OR “TKA” Or “Total Knee Replacement” Or “TKR” AND “Venous Thromboembolism” OR “VTE” OR “Deep Vein Thrombosis” OR “DVT” OR “Thrombosis” OR “Pulmonary Embolism” OR “Anticoagulation”

### Eligibility criteria and study selection

The inclusion criteria for the meta-analysis were as follows: (1) level I and II randomised clinical trials (RCT) and prospective cohort studies (PCS); (2) written in or translated into the English language; (3) patients of Asian demographic; (4) incidence of VTE following TKA reported; (5) minimum number of ten patients. Case reports, review articles, published abstracts, studies involving fewer than ten patients, and duplicate data were excluded from this review. Articles not written in English, or where access to the full text was unavailable, were also excluded.

The above criteria were applied to the 71 articles that were initially screened; 57 articles were excluded as they were not full-text articles, contained non-Asian patients, or were not RCTs or PCS. Fourteen articles of RCTs and PCS were analysed, totalling 4563 patients. Three groups totalling 304 patients in the selected studies were excluded from analysis for the following reasons: (i) 100 patients who received no chemoprophylaxis and had undetermined rates of mechanical prophylaxis and 72 patients who received no chemoprophylaxis with 90.3% rate of mechanical prophylaxis were excluded to reduce bias as they could not be classified definitively as no prophylaxis or mechanical-only prophylaxis [14]; (ii) 74 patients on Fondaparinux and 49 patients on Indomethacin from two separate studies were excluded as they could not be classified as LMWH and had a sample size too small to be compared with sufficient statistical power [15, 16]; (iii) 9 patients on Aspirin from a single study were excluded as the sample size was too small to be of any statistical value [14]. The total number of patients used for network meta-analysis following exclusion totalled 4259.

Seven systematic reviews and meta-analyses were analysed, and none was found to be a duplicate of this study.

### Data collection and statistical analysis

All data from the texts, figures and tables of the included studies were extracted to Microsoft Excel spreadsheet software for analysis and review. The specific information extracted included the following: (i) study details, including study design and level of evidence, (ii) study population details, including number of patients, (iii) objective of study, (iv) type of VTE prophylaxis used, (v) modality of VTE diagnosis, (vi) number of VTEs reported and (vii) incidence of minor and major bleeding.

**Fig. 2:**
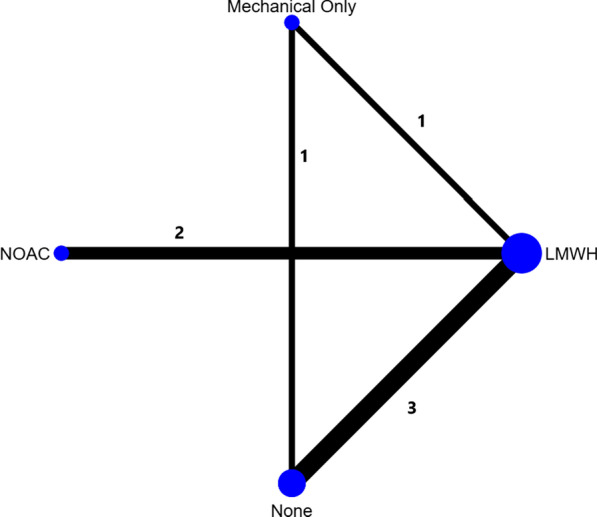
Network plot representing trial arms in the studies used in the network meta-analysis

Outcome data were stratified according to prophylaxis utilised. The categories used were no prophylaxis (NP), mechanical-only prophylaxis (MOP), low-molecular-weight heparin (LMWH) and NOACs. Those on LMWH and NOACs were placed into their groups irrespective of whether they received simultaneous mechanical prophylaxis as these data were not routinely available in all studies. A network plot was created to compare the trial arms of the studies, as shown in Fig. 2.

All statistical analyses were performed using STATA software and Cochrane Revman 5.3 Software (Cochrane Collaboration 2014). A 95% confidence interval was used to calculate the risk ratio for this study. A *p* value of < 0.05 was considered significant.

### Risk of bias assessment of studies

The Cochrane Risk of Bias (ROB) Tool 2 was used to stratify RCT Risk of Bias, and the ROBIN-I Tool was used to stratify risk of bias in PCS as shown in Table 2 [13].

**Table 2:**
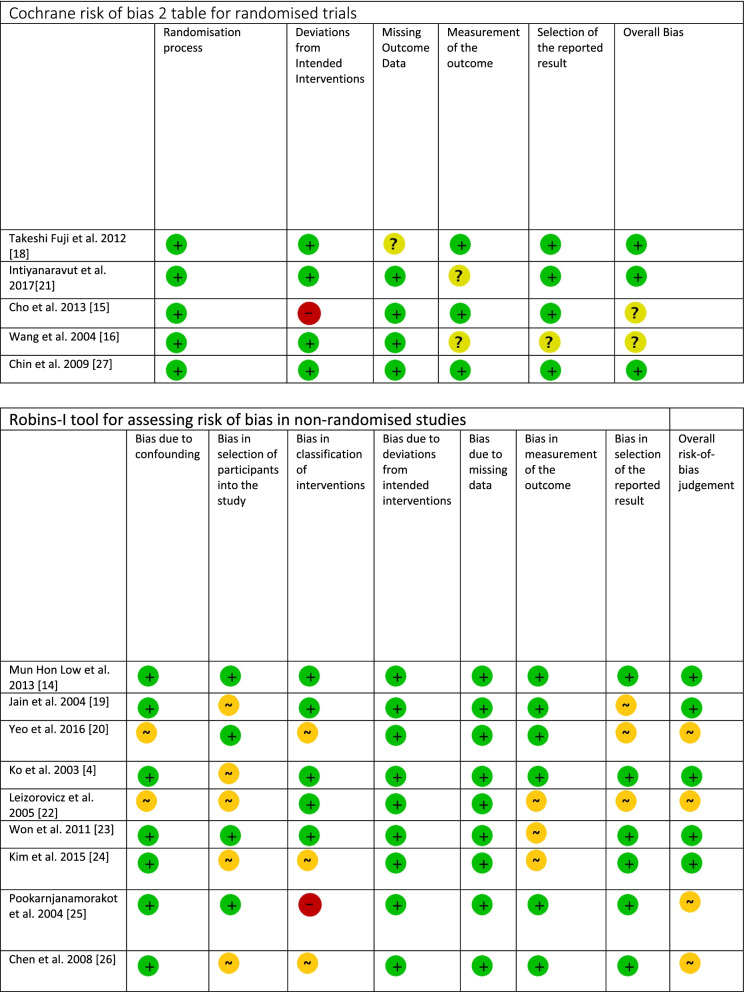
Summary of bias in included trials

Risk of Bias 2 legend: +, low risk; ?, uncertain: −, high risk

Robins-I Tool: +, low risk, ~, moderate risk; −, serious risk; ×, critical risk

### Quality assessment of studies

The Grading of Recommendations Assessment, Development and Evaluation (GRADE) system was used to assess the quality of the studies. The five domains of risk of bias, imprecision, inconsistency, indirectness and publication bias were ranked. These were ranked on the basis of our take on the certainty of evidence, with high being the most certain and very low being the least certain, as seen in Table 3 [17].

**Table 3:** Grade system evaluation for the quality of evidence

Number of studies	Risk of bias	Imprecision	Inconsistency	Indirectness	Publication bias
Primary outcomes					
VTE incidence (ultrasound)—7	L	L	L	M	VL
VTE incidence (venography)—5	M	M	M	M	L
VTE incidence (symptomatic)—8	M	H	H	M	M
PE incidence—11	M	H	H	L	M
Secondary outcomes					
Minor bleeding—4	M	M	M	L	L
Major bleeding—6	M	H	H	M	L

H, high certainty; M, moderate certainty; L, low certainty; VL, very low certainty

## Results

The 14 articles assessed in this systematic review and network meta-analysis included a total of 4259 patients [4, 14–16, 18–27]. Characteristics of the included studies are presented in Table 4. Most studies utilised similar exclusion criteria. This included current anti-coagulant use, previous VTEs, cardiovascular disease, haemophilia or thrombotic tendencies, recent surgeries, gastrointestinal bleeding, renal and liver impairment, and malignancy.

**Table 4:** Study characteristics and details

	Prophylaxis type	Reported primary outcomes	Reported secondary outcomes
Mun Hon Low et al. 2013 [14]	LMWH (*n* = 72) NOAC (*n* = 15)	US	Major
Takeshi Fuji et al. 2012 [18]	LMWH (*n* = 66) NOAC (*n* = 152)	V, C	Minor, major
Jain et al. 2004 [19]	NP (*n* = 26)	US, C	None
Yeo et al. 2016 [20]	MOP (*n* = 802)	C	None
Ko et al. 2003[4]	NP (*n* = 58)	US	None
Intiyanaravut et al. 2017 [21]	LMWH (*n* = 25) NP (*n* = 25)	US, C	Minor, major
Leizorovicz et al. 2005 [22]	NP (*n* = 944)	C	Major
Cho et al. 2013 [15]	MOP (*n* = 74)	US	Minor, major
Won et al. 2011 [23]	NP (*n* = 440)	US	None
Kim et al. 2015[24]	MOP (*n* = 874)	V, US	None
Wang et al. 2004 [16]	LMWH (*n* = 50) NP (*n* = 51)	V, C	None
Pookarnjanamorakot et al. 2004 [25]	NP (*n* = 67)	V, C	None
Chen et al. 2008 [26]	NP (*n* = 78)	V, C	None
Chin et al. 2009 [27]	LMWH (*n* = 110) MOP (*n* = 220) NP (*n* = 110)	U	Minor, major

V, venography; US, ultrasound; C, clinical diagnosis; major, major bleeding; minor, minor bleeding

### NOACs

Darexaban and rivaroxaban were the only two NOACs that were utilised. Darexaban was used with dosing of 15–30 mg daily ,whilst the rivaroxaban study did not report any dosages.

### LMWHs

Of the studies that included LMWH, most patients received enoxaparin, with only one study using Fraxiparine. The dose of enoxaparin ranged from 20 mg to 40 mg for 5–10 days, while the dose for Fraxiparine was 0.2–0.4 ml till the day of patient discharge.

### Mechanical-only prophylaxis (MOP)

MOP included graduated compression stockings (GCS), intermittent pneumatic compression (IPC) or both. Sole early mobilisation was not considered a type of mechanical prophylaxis owing to difficulty in standardisation. Pressures of IPC ranged from 40 mmHg to 52 mmHg.

### No prophylaxis (NP)

Studies that reported no chemoprophylaxis and no mechanical prophylaxis were classified under “no prophylaxis”.

### Primary outcome: VTE incidence

**Fig. 3:**
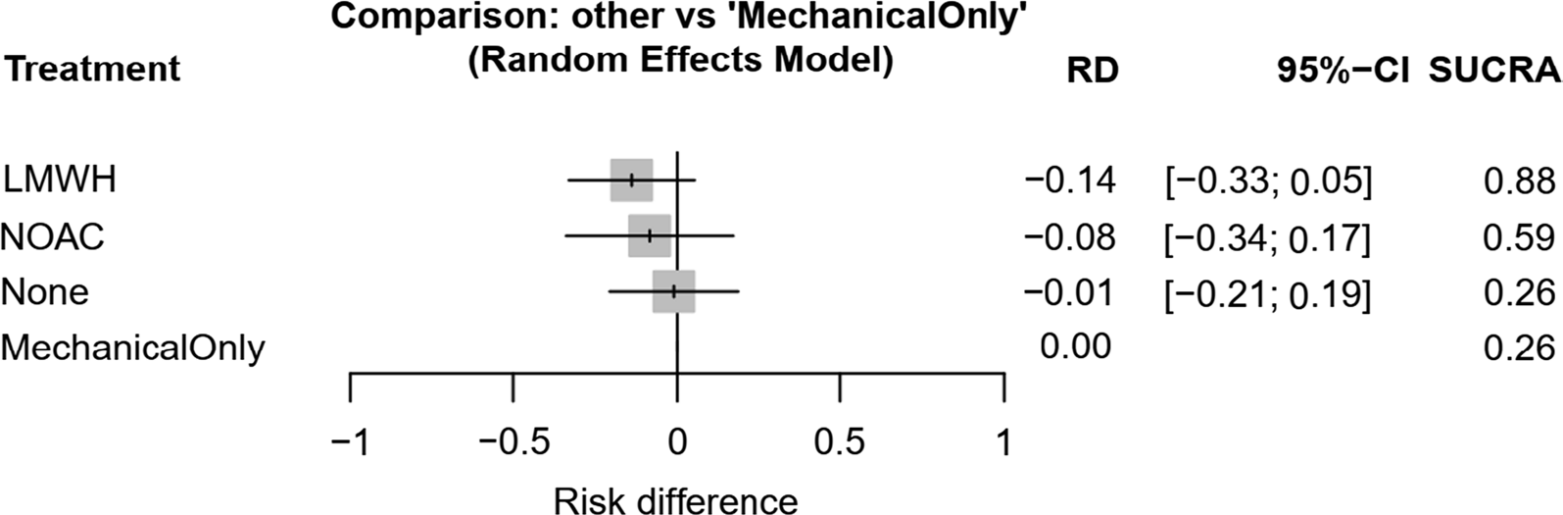
Treatment effect based on VTE incidence against mechanical-only prophylaxis

**Fig. 4:**
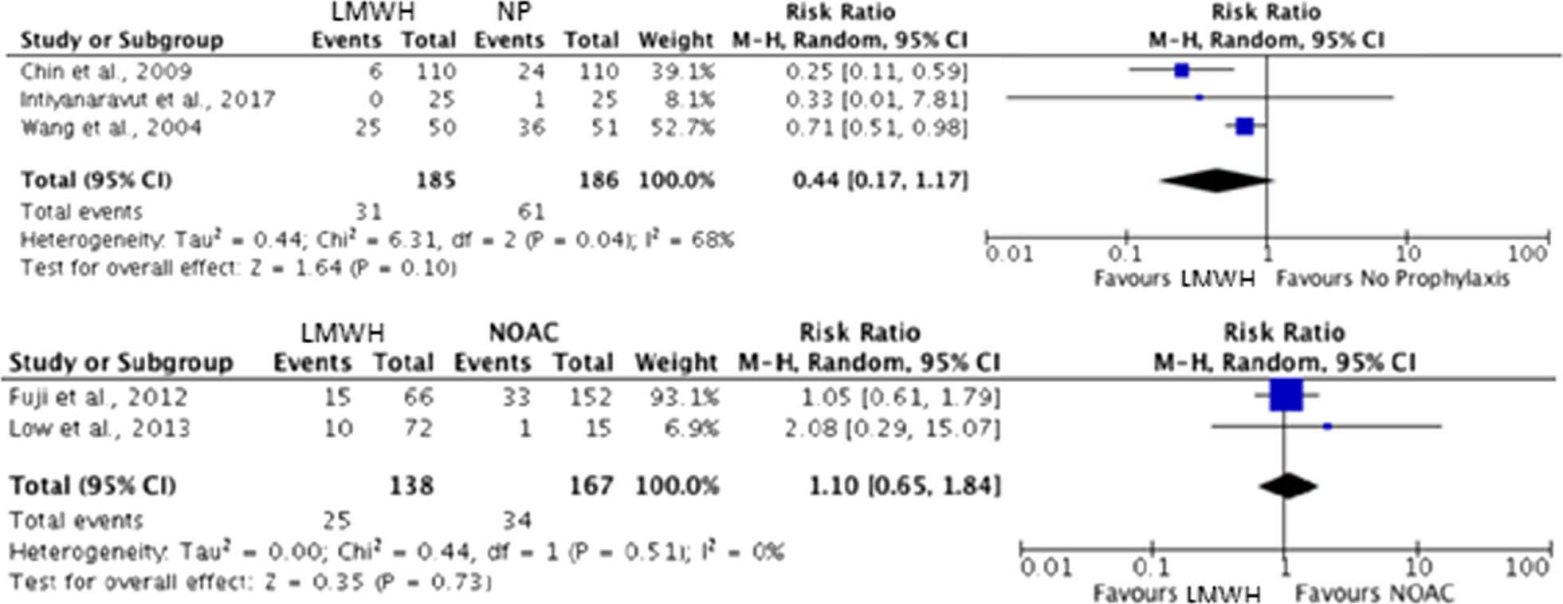
Pairwise forest comparing LMWH versus NP and LMWH versus NOACs

Incidence of VTE regardless of diagnosis method stratified by prophylaxis type was: NOAC (20.36%), LMWH (17.34%), NP (8.78%) and MOP (7.26%). A ranked forest plot (Fig. 3) with confidence intervals was generated to determine the effectiveness of no prophylaxis, MOP, LMWH and NOACs. A pairwise forest plot (Fig. 4) was also created to compare the efficacy of prophylaxis types between trials. Unfortunately, owing to a lack of studies with multiple trial arms, only comparisons between LMWH and NP and LMWH and NOACs could be analysed. Neither analysis identified statistically significant differences in VTE incidence.

To reduce bias, instead of pooling values, the incidences were further grouped according to the modality of diagnosis: ultrasound, venography and clinically diagnosed VTEs.

**Fig. 5:**
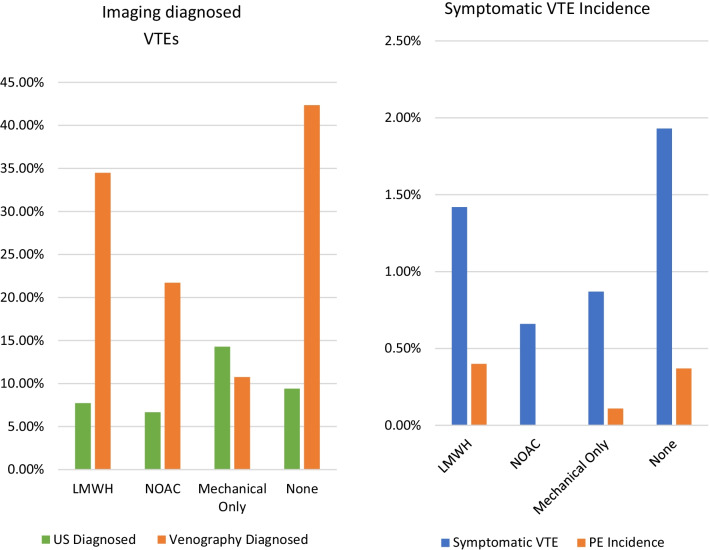
Graphical representations of VTE incidence accounting for diagnosis modality

Ultrasound (US) imaging was utilised in seven studies (1175 patients), with three studies (585 patients) using Duplex and four studies (590 patients) using Doppler. Incidence of VTE diagnosed by US, stratified by prophylaxis type, was: MOP (14.29%), NP (9.41%), LMWH (7.73%) and NOAC (6.67%) (Fig. 5, Table 5). MOP had a statistically higher ultrasound-diagnosed VTE incidence than LMWH (6.56%, *p* = 0.024) and NP (4.88%, *p* = 0.026). We did not observe a statistical difference between LMWH and NOACs.

**Table 5:** Incidence of VTE accounting for diagnosis type with statistical analysis

VTE incidence by prophylaxis and diagnosis type				
Prophylaxis type	Ultrasound VTE incidence	Venography VTE incidence	Symptomatic VTE incidence	Pulmonary embolism incidence
LMWH	16/207, 7.73%	40/116, 34.48%	2/141, 1.42%	1/251, 0.40%
NOAC	1/15, 6.67%	33/152, 21.71%	1/152, 0.66%	0/152, 0.00%
MOP	42/294, 14.29%	94/874, 10.76%	7/802, 0.87%	2/1896, 0.11%
NP	62/659, 9.41%	83/196, 42.35%	23/1191, 1.93%	5/1359, 0.37%

Statistical analysis				
LMWH versus NOAC	*p* = 0.881	*p* = 0.020	*p* = 0.516	*p* = 0.435
LMWH versus MOP	*p* = 0.024	*p* < 0.001	*p* = 0.542	*p* = 0.242
LMWH versus NP	*p* = 0.460	*p* = 0.171	*p* = 0.674	*p* = 0.944
NOAC versus MOP	*p* = 0.407	*p* < 0.001	*p* = 0.787	*p* = 0.690
NOAC versus NP	*p* = 0.719	*p* < 0.001	*p* = 0.263	*p* = 0.453
MOP versus NP	*p* = 0.026	*p* < 0.001	*p* = 0.057	*p* = 0.254

Venography was used in five studies (1338 patients). Two studies (941 patients) utilised radionuclide venography, while the three other studies (397 patients) did not report venography type. Venography-diagnosed VTE incidence by prophylaxis type was as follows: NP (42.35%), LMWH (34.48%), NOAC (21.71%) and MOP (10.76%) (Fig. 5, Table 5). MOP had significantly lower VTE incidence compared with LMWH (23.72%, *p* < 0.001), NOACs (10.95%, *p* < 0.001) and NP (31.59%, *p* < 0.001). NOACs showed superiority over LMWH (12.77%, *p* = 0.02) and NP (20.64%, *p* < 0.001) at reducing venography-diagnosed VTEs but was still inferior to MOP, as described above. There was no significant benefit to utilising LMWH over NP.

Symptomatic VTE incidence was reported in eight studies with a total of 2286 patients and incidence of 1.44%. Reported symptomatic incidence of VTE stratified by prophylaxis type was as follows: NP (1.93%), LMWH (1.42%), MOP (0.87%) and NOAC (0.66%) (Fig. 5, Table 5). There were no statistical differences between the prophylaxis types.

Incidence of pulmonary embolism was reported in 11 studies (3658 patients) and was found to be 0.22%. PE incidence stratified by prophylaxis type was as follows: LMWH (0.40%), NP (0.37%), MOP (0.11%) and NOAC (0.00%) (Fig. 5, Table 5). There were no statistical differences between the prophylaxis types.

None of the studies reported any VTE-related deaths following TKA.

### Secondary outcome: bleeding incidences

**Fig. 6:**
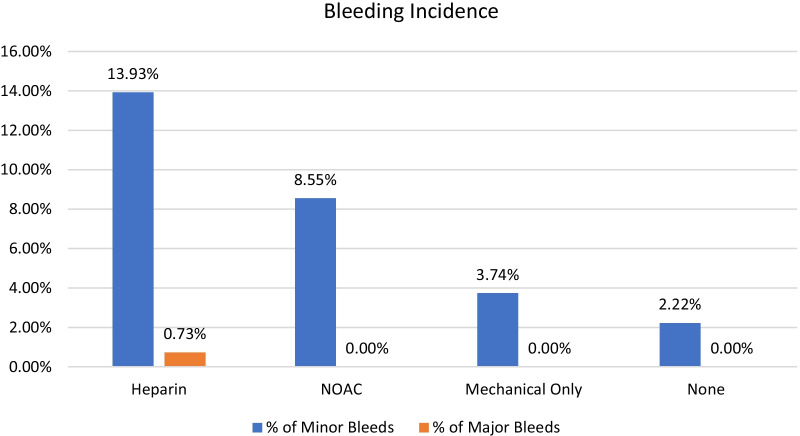
Graphical representation of bleeding incidence

Four studies with a total of 782 patients reported minor bleeding. Minor bleeding incidence according to prophylaxis type was as follows: LMWH (13.93%), NOAC (8.55%), MOP (3.74%) and NP (2.22%) (Table 6). LMWH had a significantly higher incidence of minor bleeding than MOP (10.19%, *p* < 0.001) and NP (11.71%, *p* < 0.001) (Fig. 6, Table 6). Similarly, NOACs had a significantly higher incidence of minor bleeding than MOP (4.81%, *p* < 0.33) and NP (6.33%, *p* < 0.02). The difference in incidence of minor bleeding between LMWH and NOACs was not significant.

**Table 6:** Bleeding incidence with statistical analysis

Bleeding incidence		
Prophylaxis type	Minor bleeding	Major bleeding
LMWH	28/201, 13.93%	2/273, 0.73%
NOAC	13/152, 8.55%	0/152, 0.00%
MOP	11/294, 3.74%	0/294, 0.00%
NP	3/135, 2.22%	0/135, 0.00%

Statistical analysis		
LMWH versus NOAC	*p* = 0.119	*p* = 0.289
LMWH versus MOP	*p* = < 0.001	*p* = 0.142
LMWH versus NP	*p* = < 0.001	*p* = 0.317
NOAC versus MOP	*p* = 0.033	*p* < 0.001
NOAC versus NP	*p* = 0.020	*p* < 0.001
MOP versus NP	*p* = 0.412	*p* < 0.001

Six studies with a total of 1813 patients reported major bleeding. Major bleeding incidence according to prophylaxis type was as follows: LMWH (0.73%) and NOACs, MOP and NP (0%) (Fig. 6, Table 6). The only two incidences of major bleeding were associated with LMWH usage. However, this incidence of major bleeding in LMWH compared with the other prophylaxis types was not statistically significant. We observed that NOACs had the same major bleeding incidence as MOP and NP.

## Discussion

This systematic review and network meta-analysis was designed to evaluate the best prophylaxis strategy against VTEs following total knee arthroplasty in Asian populations, for which effectiveness was measured in both VTE incidence and bleeding incidence. Apparent efficacy of each prophylaxis group in reducing VTE incidence varied with diagnosis type, with varying results for symptomatic, venography-diagnosed and ultrasound-diagnosed VTE incidence. However, we observed that there was no statistically significant superiority of chemoprophylaxis nor mechanical prophylaxis against no prophylaxis in reducing symptomatic VTE incidence, PEs or deaths. Regarding bleeding incidence, we observed that minor bleeding incidence was higher in LMWH and NOACs than in MOP and NP. There was no significant difference in major bleeding across all prophylaxis types. Our recommendations and analysis of the results will be explored below.

Current literature for post-TKA VTE prophylaxis does not present clear evidence as to which prophylaxis type is optimal in Asian populations. We observed that apparent efficacy of the various prophylaxis types was not necessarily agreed upon by across the various diagnostic modalities. A discrepancy we observed was ultrasound diagnosis showing LMWH superiority over MOP, clinical diagnosis showing no significant differences and venography diagnosis showing MOP superiority over LMWH. Some of these discrepancies were also found in current published literature. Two ultrasound-based studies completed in Asian populations by Zhang et al. [28] and Chin et al. [27] showed decreased VTE incidence in chemoprophylaxis against MOP, which conflicted with one of the largest Asian epidemiological studies conducted by Yhim et al. [29], who found a higher incidence of symptomatic VTE in patients receiving chemoprophylaxis compared with patients receiving no chemoprophylaxis and undetermined amounts of MOP. We were unable to find established literature comparing venography-diagnosed MOP and LMWH. It is important for clinicians to be mindful of the desired outcomes when choosing a prophylaxis regimen in view of diagnosis modality affecting apparent efficacies. This would largely be influenced by the availability of diagnostic modalities in the institution.

We observed significantly higher ultrasound-diagnosed VTE incidence in MOP compared with NP, which contrasts with existing literature in both Asian and Western populations that has extensively proven that mechanical prophylaxis following TKAs is effective at reducing VTE incidence [10, 11, 27, 30]. Our contradictory findings are likely a result of compilation of ultrasonography results of varying unreported methodologies. It is well documented that the sensitivity and specificity for ultrasonography is dependent on patient type, user and area scanned with agreement for venography and ultrasonography ranging from 92.9% (proximal vessels) to 60% (distal vessels) [31]. As such, we advise that datasets combining ultrasonography data be interpreted with caution. For the above reasons, venography results of this study will be prioritised over ultrasound-diagnosed results.

It is notable that MOP was significantly more effective than LMWH and NOACs, with undetermined rates of mechanical prophylaxis in reducing venography-diagnosed VTE incidence. This is potentially due to the effectiveness of MOP at preventing VTE such that the LMWH and NOAC subgroups without 100% concurrent mechanical prophylaxis usage had a reduced apparent effectiveness. This is supported by a retrospective study by Loh et al. [32], who found no statistical difference in US-diagnosed DVT incidence in MOP against concurrent chemical and mechanical prophylaxis usage. As such, mechanical prophylaxis should certainly be considered in all patients after TKA in view of its known benefits with no added bleeding incidence. However, routine chemoprophylaxis for low-risk patients may not be necessary. Decision to prescribe chemoprophylaxis should be individualised to patients with a higher risk of developing VTE with no added observed incidence of major bleeding.

NOACs, especially rivaroxaban and dabigatran, have become increasingly utilised post-TKA owing to their fixed dosages and the lack of monitoring requirements [33]. We observed NOACs to be more effective than LMWH in reducing venography-diagnosed VTE. Yhim et al. [29] found superiority of rivaroxaban over LMWH in reducing symptomatic VTEs, with similar findings found in Western populations [34]. However, dabigatran, the only other NOAC to be trialled in large-scale studies, has a similar efficacy profile to LMWH in reducing venography and symptomatic VTE incidence [35, 36]. We observed no significant differences in minor or major bleeding incidence in NOACs and LMWH, which is also supported by current literature [34, 37].

Symptomatic VTE and major bleeding incidence are arguably the two most important factors to consider when selecting a VTE-prophylaxis regime. The significance of DVT in the calf is not entirely clear, and its treatment is controversial. The routine use of chemoprophylaxis is not without its risks. Chin et al. [27] reported an 11% incidence of bleeding complications with use of enoxaparin. These complications would have an undesirable effect on post-operative rehabilitation as well as length of stay. Ultimately, healthcare costs and utilisation will increase. We did not observe any significant differences between prophylaxis types in terms of symptomatic VTE and major bleeding incidence. The literature on routine chemoprophylaxis in Asian populations is divided, with Fuji et al. [18] and Intiyanaravut et al. [21] not observing any significant differences in symptomatic VTE incidence between routine chemoprophylaxis (darexaban and enoxaparin) and placebo chemoprophylaxis or NP. This is in contrast to the findings of Wang et al. [16], who observed significantly higher symptomatic DVTs in patients administered placebo than in those given indomethacin and enoxaparin. For major bleeding, current literature suggests that there are no significant differences in major bleeding incidence regardless of prophylaxis type, which is in accordance with our findings [18, 21, 27]. To the best of our knowledge, there are currently no trials reporting a significantly higher incidence of major bleeding in any prophylaxis type following TKA in Asian populations.

There are two main limitations to using evidence-based medicine in this space of thromboprophylaxis following total knee arthroplasty in Asian populations. Firstly, the modality of diagnosis changes the apparent efficacy of one prophylaxis type over another, meaning that clinicians should be aware of what outcomes they are trying to achieve when choosing a prophylaxis strategy. Secondly, despite statistically significant findings in venography and ultrasonography, there were no differences in symptomatic VTE or major bleeding incidence, which are the most important factors to consider when choosing a prophylaxis regime. As such, an evidence-based approach with patient centricity may prove to be an effective strategy in managing post-TKA VTE prophylaxis in Asian populations.

With a recent shift towards early mobilisation and enhanced recovery after TKA as an effective method of VTE prophylaxis, routine chemoprophylaxis especially in Asian populations has become an ever-increasing topic of debate [38]. A recent study in France demonstrated that using an enhanced recovery after surgery (ERAS) regime alongside routine LMWH or DOAC usage resulted in a higher incidence (1.7%) of major bleeding compared with symptomatic VTE following THA and TKAs, where ERAS comprised getting out of bed within 24 h post-operation and early discharge [39]. In Asian populations who are inherently less prone to VTE this is particularly relevant, and further studies into the effectiveness of ERAS in Asians are likely to be of clinical value.

The main strength of this systematic review and network meta-analysis was the stratification of results by using the modality of diagnosis instead of using a pooled VTE incidence to reduce heterogeneity and variation. However, this study does come with limitations. Firstly, most studies used a low-risk population with minimal co-morbidities, leading to data that may not be generalisable, thus affecting external validity. A lack of specific details included in trials was another limitation. We could not analyse the frequency of mechanical prophylaxis used in patients with concurrent chemoprophylaxis, specific types of mechanical prophylaxis used, and specific effects of the individual ethnicities of Asian patients. To acquire samples of sufficient size, multiple generic classifications needed to be used, including mechanical prophylaxis, LMWH and NOACs, which may not be representative of the efficacy of the individual subtypes. Lastly, we found contrasting results between venography and ultrasonography, which is likely the result of varied methodology as studies were pooled in this study. However, as venography is the gold standard of DVT diagnosis, the venography results were prioritised over ultrasonography.

## Conclusion

VTE prophylaxis following TKAs in Asians has remained a controversial topic of debate owing to the lower inherent risk of VTE in Asians compared with Western populations. This systematic review and network meta-analysis has shown that mechanical prophylaxis in the form of GCS or IPC significantly reduces venography-diagnosed VTE incidence over having no prophylaxis. Interestingly, we did find that patients on either LMWH or NOAC with undetermined amounts of mechanical prophylaxis had significantly higher incidences of venography-diagnosed VTE than patients using mechanical prophylaxis in isolation, which may be a testament to the effectiveness of mechanical prophylaxis. Chemoprophylaxis in the form of LMWH or NOACs, as expected, led to an increased incidence of minor bleeding and, in view of the effectiveness of mechanical-only prophylaxis and low rates of VTE in Asians, may not be indicated for routine use in all patients. However if chemoprophylaxis is warranted, NOACs are superior to LMWH at reducing venography-diagnosed VTE incidence, with no increase in bleeding incidents. Overall, an evidence-based approach with patient centricity may prove to be an effective strategy in managing post-TKA VTE prophylaxis in Asian populations. We recommend mechanical prophylaxis to be used routinely while reserving chemoprophylaxis, with the preferred agent of choice being NOACs, for patients deemed at high risk of VTE.

## Data Availability

All data and materials used has been sourced are available to public.
